# Evaluation of Mechanical and Interfacial Properties of Bio-Composites Based on Poly(Lactic Acid) with Natural Cellulose Fibers

**DOI:** 10.3390/ijms20040960

**Published:** 2019-02-22

**Authors:** Laura Aliotta, Vito Gigante, Maria Beatrice Coltelli, Patrizia Cinelli, Andrea Lazzeri

**Affiliations:** 1 Department of Civil and Industrial Engineering, University of Pisa, Via Diotisalvi, 2, 56122 Pisa, Italy; laura.aliotta@dici.unipi.it (L.A.); vito.gigante@dici.unipi.it (V.G.); maria.beatrice.coltelli@unipi.it (M.B.C.); 2 Interuniversity National Consortium of Materials Science and Technology (INSTM), Via Giusti 9, 50121 Florence, Italy

**Keywords:** bio-composites, mechanical properties, poly(lactic acid), cellulose fibers

## Abstract

The circular economy policy and the interest for sustainable material are inducing a constant expansion of the bio-composites market. The opportunity of using natural fibers in bio-based and biodegradable polymeric matrices, derived from industrial and/or agricultural waste, represents a stimulating challenge in the replacement of traditional composites based on fossil sources. The coupling of bioplastics with natural fibers in order to lower costs and promote degradability is one of the primary objectives of research, above all in the packaging and agricultural sectors where large amounts of non-recyclable plastics are generated, inducing a serious problem for plastic disposal and potential accumulation in the environment. Among biopolymers, poly(lactic acid) (PLA) is one of the most used compostable, bio-based polymeric matrices, since it exhibits process ability and mechanical properties compatible with a wide range of applications. In this study, two types of cellulosic fibers were processed with PLA in order to obtain bio-composites with different percentages of microfibers (5%, 10%, 20%). The mechanical properties were evaluated (tensile and impact test), and analytical models were applied in order to estimate the adhesion between matrix and fibers and to predict the material’s stiffness. Understanding these properties is of particular importance in order to be able to tune and project the final characteristics of bio-composites.

## 1. Introduction

The increasing environmental awareness coupled with the circular economy policy, supported by new regulations, are driving plastic industries as well as consumers toward the selection of ecologically friendly raw materials for their plastic products. Several products developed for large application fields are based on natural fibers in composites with a polypropylene matrix [1]. These materials are not compostable and are hardly recyclable; thus, work is in progress to investigate new composites with biopolymers as polymeric matrices (bio-composites), offering the advantage of bio-recycling options at the end of their service life through composting or anaerobic digestion. In this contest, bio-based polymers reinforced with natural fibers are beneficial to prepare biodegradable composite materials [2]. The most used biopolymers for this application are poly(lactic acid) (PLA), cellulose esters, polyhydroxyalkanoates (PHAs), and starch-based plastics [3,4].

In applications where biodegradability offers clear advantages for customers and the environment, such as single-use applications (packaging and agriculture), it is expected that the demand for these biopolymers will increase [5,6,7].

In this context, poly(lactic acid) (PLA) is certainly one of the best candidates, being compostable and produced from renewable resources such as sugar beets or corn starch [8,9]. In addition to its biodegradability and renewability, PLA exhibits at room temperature a Young’s modulus of about 3 GPa, a tensile strength between 50 and 70 MPa with an elongation at break of about 4%, and an impact strength close to 2.5 kJ/m^2^ [10].

Although PLA is considered a sustainable alternative to traditional petroleum-based plastics, many drawbacks must be overcome in order to enlarge its application field. In particular, PLA has a relatively higher cost (2.5–3.0 Euro/Kg) compared to commodity petro-derived polymers (1 Euro/Kg), it has low flexibility, bad impact resistance, low thermal stability (due to its high glass transition temperature, T_g_ ≈ 60 °C), and low crystallization rates that could limit its applications [11].

Generally, composite materials show enhanced mechanical and physical properties when compared to their individual composite components [12,13]. However, especially when the fibers are very short and randomly oriented, the resulting composite does not necessarily provide enhanced properties. In this case, the benefit of composite production is envisaged in cost savings, lighter weight, and promoted degradability.

The study of the interaction between the fiber and the polymeric matrix in a composite plays an important role because it influences both physical and mechanical properties of the final materials. In particular, the adhesion—that is the ability to transfer stresses across the interface—is often related to a combination of different factors such as the interface thickness, the interphase layer, the adhesion strength, and the surface energy of the fibers [14,15,16].

Natural fibers have many advantages compared to synthetic ones. They are recyclable, biodegradable, renewable, have relatively high strength and stiffness, and do not cause skin irritation [17]. On the other hand, there are also some disadvantages such as moisture uptake, the presence of color, the presence of odor when heated or burned during processing, quality variations, and low thermal stability. Many investigations have been carried out on the potential of natural fibers as reinforcement for composites, and in several cases the results have shown that natural fiber composites reached a good stiffness, but their final strength was not improved [18,19].

Composite manufacturing industries are looking for plant-based natural fiber reinforcements, such as flax, hemp, jute, sisal, kenaf, and banana as alternative materials to replace synthetic fibers. Lignocellulose fibers have also been considered for replacing glass fibers [20] as lignocellulose fibers are cheaper, lighter than glass fibers, and safer to be handled by workers [21].

Due to their advantages of low cost, biodegradability, large availability, and valuable mechanical and physical properties [22], a wide variety of lignocellulose fibers and natural fillers—coming from agricultural and industrial crops such as corn, wheat, bagasse, orange and apple peel algae, and sea grasses-derived fibers—have been used in the production of composites in various industrial sectors, such as packaging, automotive industry, and building [23,24,25,26].

For these reasons, several bio-composites were produced with a polymeric biodegradable matrix such as PLA and natural fibers. Tserki et al. [27] investigated the usefulness of lignocellulose waste flours derived from spruce, olive husks, and paper flours as potential reinforcements for the preparation of cost-effective bio-composites using PLA as the matrix. Petinakis et al. [28] studied the effect of wood flour content on the mechanical properties and fracture behavior of PLA/wood flour composites. In several natural fiber bio-composites with PLA as the matrix, the interfacial adhesion between the polymeric matrix and the fibers was poor [29]. Thus, the incorporation of lignocellulose materials into biodegradable polymer matrices, such as PLA, generally has the effect of improving the mechanical properties, such as tensile modulus, but sometimes the strength and toughness of these bio-composites are not improved.

Although several reviews [30,31,32,33] deal with lignocellulose-based composites including preparation methods and properties, most of them do not consider a deep analysis of interfacial adhesion between fiber and matrix or the application of mathematical models to explain them, which are very useful for predicting and tuning the properties of bio-composites. Thus, work remains to be done on the collective analysis of various applications of cellulose-based material. Natural fibers contain large amount of cellulose, hemicelluloses, lignin, and pectin, tending to be polar and hydrophilic, while polymeric materials are generally not polar and exhibit significant hydrophobicity [34]. The weak interfacial bonding between highly polar natural fibers and a non-polar organophilic matrix can lead to the worsening of the final properties of the bio-composites, ultimately hindering their industrial usage. Different strategies have been applied to eliminate this deficiency in compatibility and interfacial bond strength, including the use of surface modification techniques [35].

The hydrophilic nature of natural fibers decreases their adhesion to a hydrophobic matrix and, as a result, it may cause a loss of strength. To prevent this, the fiber surface may be modified in order to promote adhesion. Several methods have been proposed to modify natural fibers’ surface, such as graft copolymerization of monomers onto the fiber surface and the use of maleic anhydride copolymers, alkyl succinic anhydride, stearic acid, etc. [36].

In this work, different amounts of two types of short cellulosic fibers (with different aspect ratios) added in a PLA polymeric matrix were investigated to evaluate the final effect on the mechanical properties. Furthermore, in order to have an estimation of the matrix/fiber adhesion, the B parameter calculated from the Pukanszky’s model [37] was determined. The increase in stiffness of the final composite was also investigated using different analytical models existing in the literature with the aim to find the best fit with experimental data.

## 2. Results and Discussion

**Figure 1:**
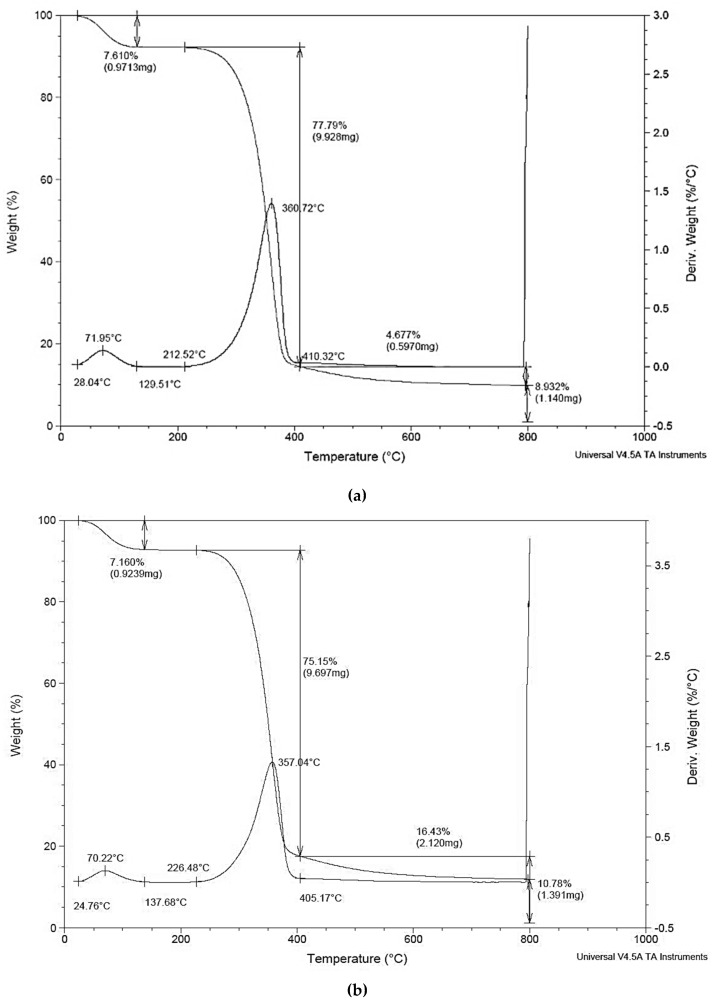
Thermogravimetric analysis (TGA) graphs of: (**a**) Arbocel^®^ 600BE/PU and (**b**) Arbocel^®^ BWW40.

Results of the thermal gravimetric analysis are reported in Figure 1. From the weight loss peaks in the weight-to-temperature graph, it is evident that the fibers will not degrade during extrusion and injection molding, since the maximum temperature reached during processing is similar to the extrusion temperature, that was equal to 190 °C. This is an advantage of cellulose fibers versus other natural fibers which very often present thermal degradation during processing with negative effects on color and odor of the produced bio-composites.

In the graph, a small weight loss at temperatures lower than 100 °C can be observed and attributed to the loss of the residual moisture trapped in the fibers. The degradation of the fibers occurs at relatively quite high temperatures, beyond 300 °C, well above those reached in the processing of composites. Consequently, we can expect that the fibers inside the composites are stable and are not degraded, as confirmed by the nice white color of the composites and the absence of odor.

**Figure 2:**
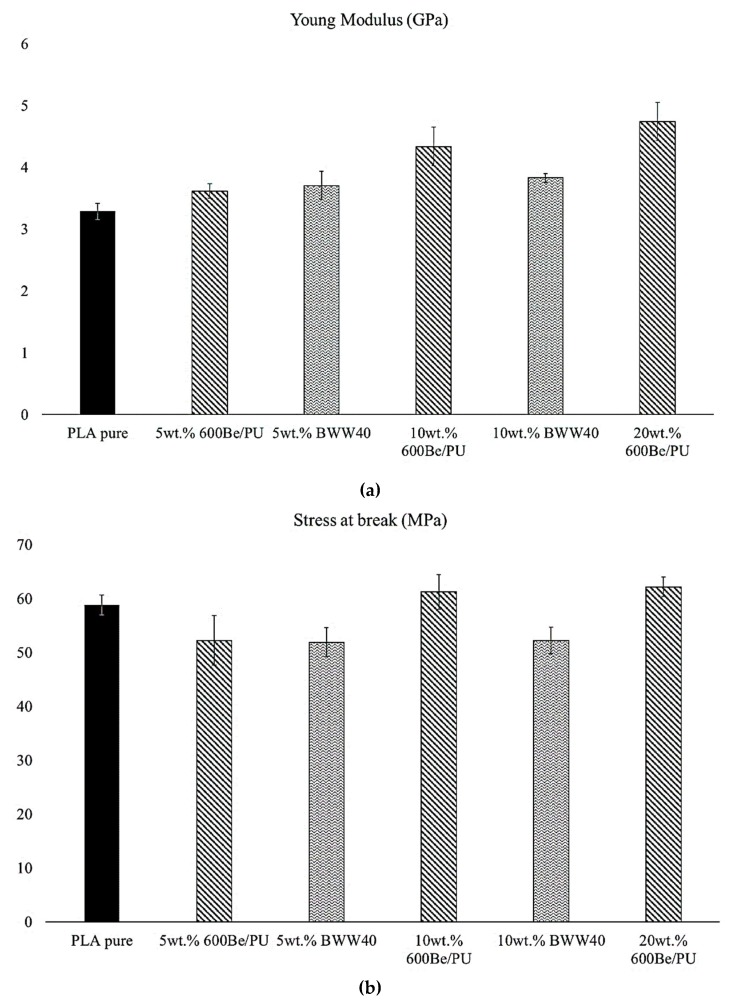

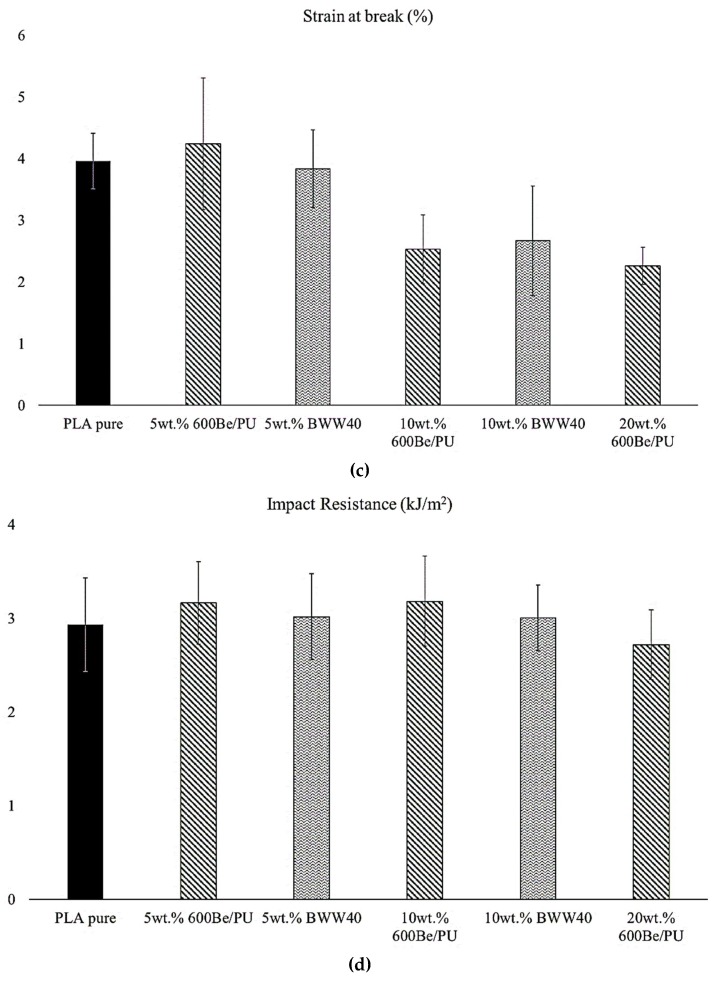
Mechanical properties of poly(lactic acid) (PLA)–Arbocel^®^ composites: (**a**) Young’s modulus, (**b**) stress at break, (**c**) strain at break, and (**d**) impact resistance.

From the results of the mechanical tests, it can be observed that, as expected, increasing the fiber content increases the elastic modulus of the composites (Figure 2a), in agreement with the trend normally observed in other studies in which cellulosic fibers were used [38] in polymeric matrices. This behavior is very common, and the stiffness increment is generally related to the higher rigidity of the reinforcement versus the polymeric matrix However, BWW40 fibers show less marked increments in the Young’s modulus compared to 600BE/PU fibers. This is likely due to the fibers’ orientation and their higher aspect ratio with respect to the former. The higher aspect ratio for BWW40 can in fact cause twisting phenomena (that in general are encountered for natural fibers [39,40]) that can influence not only the elastic modulus but also the fibers’ adhesion.

On the other hand, no significant increase in the final strength and strain at break of the composite are registered (Figure 2b,c). In particular for the composite with Arbocel^®^ BWW 40, the stress at break decreases with the filler content. Even the Charpy impact resistance does not show significant improvements (Figure 2d). From these results, we can suppose that a very little or entirely null stress transfer takes place between the fiber and the matrix, due to a lack of fibers–matrix adhesion.

**Figure 3:**
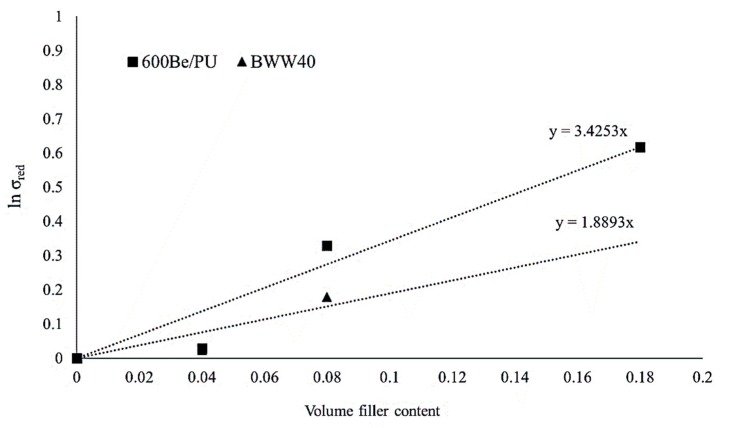
Pukánszky’s plot for PLA–Arbocel^®^ composites.

In Figure 3, the Pukánszky’s plot for the two different types of Arbocel^®^ used is reported. An approximately linear trend of *ln σ_red_* can be extrapolated to calculate the B parameter.

The values obtained for the parameter B (reported in Table 1) confirmed that the adhesion between these cellulosic fibers and the PLA matrix is very low, explaining the results of the mechanical tests in which no significant improvements in strength were observed.

In particular, for BWW40 fibers, the B parameter is lower than that of 600BE/PU fibers. This means worse adhesion that can also be related to the higher aspect ratio and possible twisting phenomena. It is therefore explained why a moderate loss in tensile strength was observed for this type of composite.

**Figure 4:**
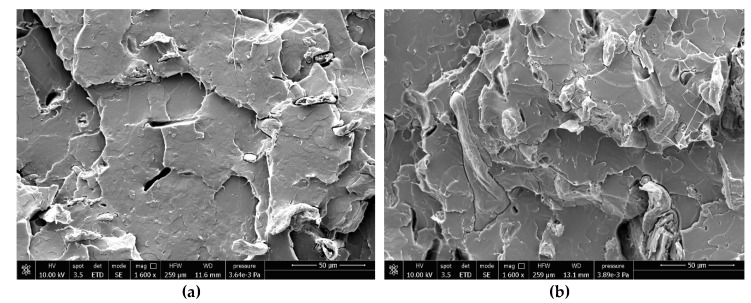
SEM micrographs of composites: (**a**) PLA + 10 wt % Be600/PU, (**b**) PLA + 10 wt % BWW40.

In Figure 4, two SEM images are displayed reporting details of both Arbocel^®^ fibers within the PLA matrix. We can observe a detachment and pull-out of the fibers due to the poor matrix–fibers adhesion.

The results obtained are consistent with those in the literature, in which a lack of adhesion was encountered in similar composite materials. The study of interfacial adhesion is, in fact, a well-known problem when natural fibers and synthetic polymers are used [4,41]. A compatibilization is necessary if we want to obtain a composite with tailored mechanical properties and good efficiency in the transferring of the stress from the matrix to the fibers.

**Figure 5:**
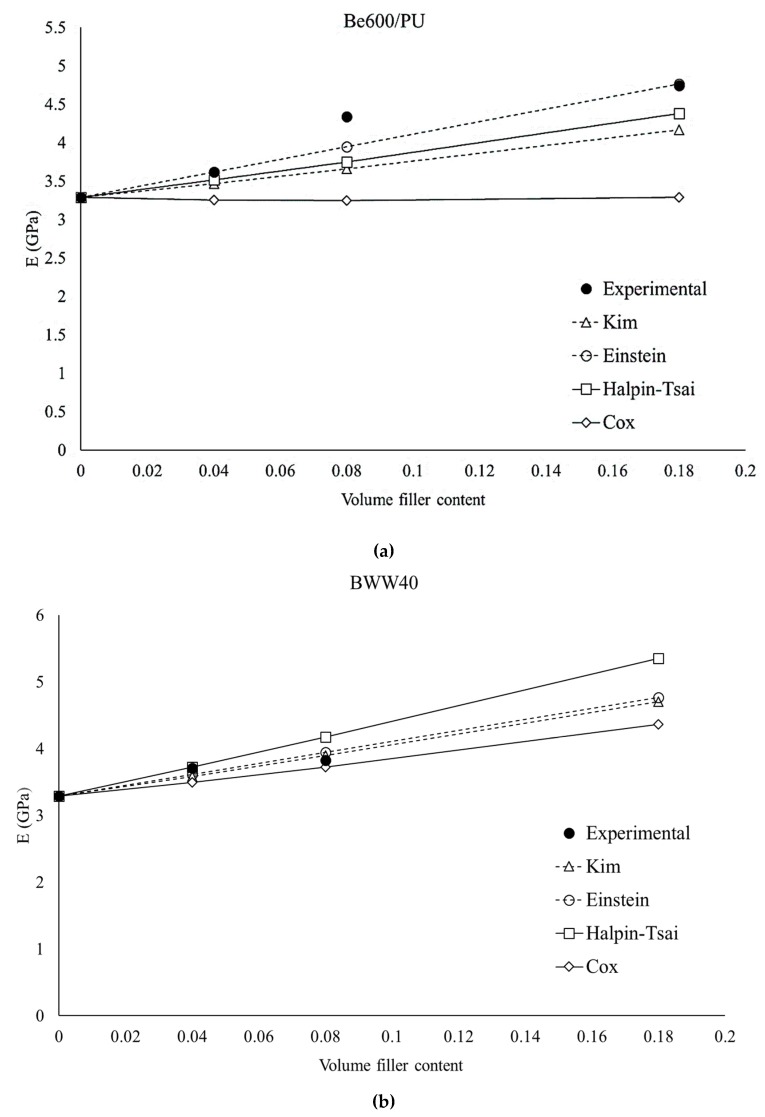
Comparison between experimental elastic modulus of the blends and mathematical models for (**a**) Be600/PU–PLA composites and (**b**) BWW40–PLA composites.

Furthermore, in this work, multiple analytical models were applied to investigate which better predicts the experimental data. The results of these analyses are reported in Figure 5.

It can be observed that the Cox model provides underestimated stiffness predictions. This is due to the fact that the Cox model is referred to as the shear lag theory in which long, straight, and discontinuous fibers completely embedded in a continuous matrix were considered [42]. Consequently, this model is not very accurate when the fibers’ aspect ratios are very small [43] and the adhesion is not good. This explains why the prediction is completely inaccurate for Be600/PU fibers, which have a very short aspect ratio (equal to 3), and improves with BWW40 fibers (aspect ratio equal to 10).

The Kim’s model derives from the shear lag theory (like the Cox model) but was extended to resolve discrepancies of the Cox model in the case of short fiber-reinforced composites. In this case, the predicted values of the elastic modulus were similar to the experimental ones [43]. Effectively, in our case, this model gives good results for both types of fiber and consequently may be efficiently applied to these systems.

The Einstein’s method is very simple and in general is applied for spheres, hence for fillers having a low aspect ratio. This model does not contain information about the geometry of the reinforcement, but the stiffness of the composites, depends only on the filler volume content [44]. Despite its simplicity, this model is able to efficiently estimate the composites’ stiffness, probably because of the fibers’ low aspect ratio.

The Halpin-Tsai equation also fits the experimental data with good accuracy. This model is often used to predict the elastic modulus of fibers that are randomly oriented on a plane [30]. In our case, the fibers are very short and not aligned. As a consequence, this model that contains an expression for the evaluation of both longitudinal and transversal moduli provides a good, but not perfect fitting.

## 3. Material and Methods

### 3.1. Materials and Characterization

The PLA used was 2003D, derived from natural resources and purchased from NatureWorks (Minnetonka, MN, USA) (grade for thermoforming and extrusion processes) [melt flow index (MFI): 6 g/10 min (210 °C, 2.16 kg), nominal average molar mass: 200,000 g/mol]. This type of PLA contains about 3–6% of D-lactic acid units in order to lower the melting point and the crystallization tendency, improving the processing ability. Two different types of commercial cellulosic short fibers, kindly provided by J Rettenmaier Sohne^®^ (Rosenberg, Germany), were used. The trade names and the main properties of these two types of fibers are:

- ARBOCEL^®^ 600BE/PU (mean diameter 20 μm, mean fiber length 60 μm, and consequently, aspect ratio 3, bulk density: 200–260 g/L, fiber density 1.44 g/cm^3^)
- ARBOCEL^®^ BWW40 (mean diameter 20 μm, mean fiber length 200 μm, and consequently, aspect ratio 10, bulk density: 110–145 g/L, fiber density 1.44 g/cm^3^)

**Figure 6:**
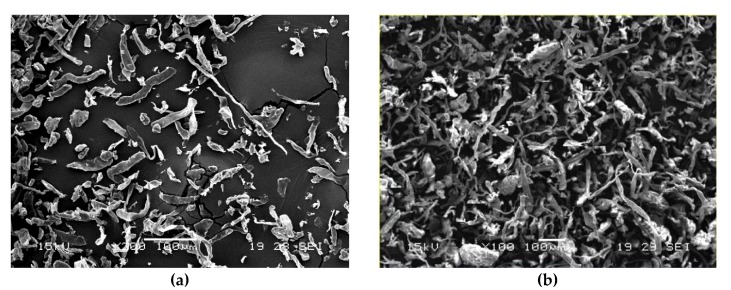
Scanning Electron Microscope (SEM) micrograph showing micro-cellulose fibers before processing: (**a**) Arbocel^®^ 600BE7PU and (**b**) Arbocel^®^ BWW40.

The morphology of these Arbocel^®^ fibers before processing is shown in Figure 6.

An increasing amount of cellulose fibers (at 5, 10, and 20 wt % corresponding to 4, 8, and 18 vol %, respectively) were added to the PLA matrix in order to produce bio-composites.

The materials, dried for at least 24 h in an air-circulated oven, were mixed in the correct quantities and then processed on a Thermo Scientific MiniLab Haake (Vreden. Germany)twin-screw extruder at a screw rate of 110 rpm/min and a cycle time of 60 s. After extrusion, the molten materials were transferred through a preheated cylinder to a Thermo Scientific Haake MiniJet II mini injection molder, for the preparation of the specimens for the Charpy and tensile tests. The Haake MiniJet II was equipped with an internal microprocessor capable of monitoring all the working parameters such as time, temperature, and injection pressure. The operative conditions of extrusion and injection molding are reported in Table 2.

It is important to observe that for the blends containing ARBOCEL^®^ BWW40, it was impossible to produce specimens containing 20 wt % of fibers because the molten material was too viscous, and the specimens were not consistent.

The tensile and impact properties of pure PLA and its composites containing different percentages of Arbocel^®^ fibers were determined.

The tensile tests were carried out at room temperature, at a crosshead speed of 10 mm/min on an Instron universal testing machine 5500R equipped with a 10 kN load cell and interfaced with a computer running MERLIN software (INSTRON version 4.42 S/N–014733H) 24 h after specimen production. At least five specimens (gauge dimensions: 25 × 5 × 1.5 mm) were tested for each sample, and the average values reported.

The impact tests were performed on V-notched specimens (width: 10 mm, length: 80 mm, thickness: 4 mm, V-notch 2 mm) using a 15 J Charpy pendulum of an Instron CEAST 9050. The standard ISO179:2000 was followed. At least 10 specimens for each blend were tested at room temperature.

The fibers and their composites were investigated by Scanning Electron Microscope (SEM) (FEI Quanta 450 FEG).

The thermogravimetric analysis (TGA) of Arbocel^®^ fibers was also performed on a TGA Rheometric Scientific at a scanning velocity of 10 °C/min from room temperature up to 1000 °C, using nitrogen as purge gas.

### 3.2. Theoretical Analysis

In this work, different analytical models were applied in order to estimate the fiber/matrix adhesion and to predict the elastic modulus of PLA–cellulose composites containing different amount of fibers.

It is well known that the strength of a composite varies on the basis of its fiber content. Adhesion between fibers and polymeric matrix has a very large effect on this property, and in particular, it was demonstrated that the reinforcement characteristics seem to have a larger effect on strength than on stiffness [45]. For rigid fillers and for fibers with a low aspect ratio (as in this study), the reinforcing effect of a filler or a fiber can be expressed quantitatively by the following equation, proposed by Pukánszky [14]:

(1) $$\sigma_{c}=\sigma_{m}\frac{1-\varphi_{f}}{1+2.5\varphi_{f}}exp\left(B\varphi_{f}\right)$$

where the terms *σ_c_* and *σ_m_*, in this case, are the tensile stress of the composite and of the matrix, respectively, *φ_f_* is the volumetric filler fraction, while the term (1 − *φ_f_*)/(1 + 2.5*φ_f_*) indicates the decreasing of the effective load-bearing cross section due to reinforcement introduction. Finally, the term exp (*Bφ_f_*) takes into account the filler–matrix interactions, by means of the interaction parameter *B* [41]. We can write Equation (1) in linear form:

(2) $$ln\left(\sigma_{red}\right)=log\frac{\sigma_{c}\left(1+2.5\varphi_{f}\right)}{\sigma_{m}\left(1-\varphi_{f}\right)}=B\varphi_{f}$$

Plotting the natural logarithm of Pukánszky’s reduced tensile strength (that is adimensional) against volume fraction (in the following graph this will be named Pukánszky’s plot) results in a linear correlation in which the linear slope is proportional to the interaction parameter *B* [37]. In this way, by applying Equation (2), it is possible to calculate the *B* parameter for the two different types of fibers and consequently obtain a simple estimation of their adhesion to the PLA matrix.

For the prediction of the elastic modulus, the present system, based on a thermoplastic matrix in which random short fibers are dispersed, is not easy to evaluate. In fact, in this case, a great number of geometric, topological, and mechanical parameters are necessary [46]. Theoretical approaches usually attempt to exploit as much readily available information (which in most cases consists of the mechanical properties of matrix and fibers and the reinforcement volume fraction) as possible, while suitable assumptions cover missing data. Referring in particular to the elastic modulus, the existing expressions can be obtained from the elasticity theory—from a sort of mixture rule—or they are simply an attempt to match theoretical curves with experimental data [42,43,44,47,48]. Some of these analytical models (reported in Table 3) consider in particular the aspect ratio, the packing factor, and the Poisson ratio, in order to better predict the elastic modulus of composites containing increasing amounts of reinforcement.

In Table 2, *Ef* and Em are the elastic modulus of the fibers and matrix, respectively, *φf* is the fibre volume fraction, *ar* is the fibers’ aspect ratio. The adimensional parameter *n* is defined as:

(3) $$\frac{2E_{m}}{E_{f}\left(1+\upsilon \right)ln\left(\frac{P}{\varphi_{f}}\right)}$$

where *υ* is the Poisson ratio of the matrix (≈ 0.4), and *P* is the fibers’ packing factor with the value 2π/√3.

Furthermore, in the Halpin-Tsai model, the two terms $E_{l}$ and $E_{t}$ are, respectively, the longitudinal and the tangential modulus, quantified by the following expressions:

(4) $$E_{l}=E_{m}\cdot \frac{1+2\cdot a_{r}\cdot \left(\frac{\frac{E_{f}}{E_{m}}-1}{\frac{E_{f}}{E_{m}}+2a_{r}}\right)\cdot \varphi_{f}}{1-\left(\frac{\frac{E_{f}}{E_{m}}-1}{\frac{E_{f}}{E_{m}}+2a_{r}}\right)\cdot \varphi_{f}}$$

(5) $$E_{t}=E_{m}\cdot \frac{1+2\cdot a_{r}\cdot \left(\frac{\frac{E_{f}}{E_{m}}-1}{\frac{E_{f}}{E_{m}}+2}\right)\cdot \varphi_{f}}{1-\left(\frac{\frac{E_{f}}{E_{m}}-1}{\frac{E_{f}}{E_{m}}+2}\right)\cdot \varphi_{f}}$$

## 4. Conclusions

In this study PLA–cellulose Arbocel^®^ fiber composites were produced and studied. Two different types of cellulose fibers having different aspect ratios were used for producing cohesive, white, nice-looking, and odorless bio-based composite materials, whose mechanical and thermal properties still meet the requirements for practical applications, such as in the packaging and agricultural sectors.

The addition of 600BE/PU cellulose fibers up to 20 wt % does not worsen the starting PLA properties (unlike with BWW 40 fibers). Consequently, 600BE/PU fibers can be used without any compatibilization in order to lower the final product cost and at the same time increase the stiffness and promote the biodegradability of the materials [20]. A compatibilization between polymeric matrix and fibers would be necessary (as verified by Pukánszky’s B parameter) if there is an interest in obtaining composites with improved tensile and Charpy impact properties with respect to those of the raw PLA-based materials, as these might be required in more demanding sectors such as automotive or electronics.

The stiffness of the composites was predicted by applying and comparing different analytical models. It was observed that a very simple model such as the Einstein’s model gives positive results. At the same time, it is not adequate to apply the Cox’s model and it is necessary to use an adjustment of it (Kim’s model). However, because of the random orientation of the fibers, the Halpin-Tsai model gives a good estimation and is preferable because it is able to provide information not only on the final composite stiffness, but also on the transversal and longitudinal composite stiffness.

## Figures and Tables

**Table 1:** B values.

Arbocel^®^ Type	B
600BE/PU	3.42
BWW40	1.89

**Table 2:** Processing conditions of Minilab and Minijet.

**Minilab**	
Extrusion temperature (°C)	190
Cycle time (s)	60
Screw rate (rpm)	110
**Minijet**	
Cylinder temperature (°C)	190
Mould temperature (°C)	60
Pressure (bar)	680
Residence time (s)	15

**Table 3:** List of the analytical expressions used in this work for the prediction of the composites’ Young’s modulus.

Model	E_composite_
Einstein	$E_{c}=E_{m}\left(1+2.5\;\varphi_{f}\right)$
Kim	$E_{c}=\varphi_{m}E_{m}+\varphi_{f}E_{f}\cdot \left\{1+\left(\sqrt{\frac{E_{m}}{E_{f}}}-1\right)\cdot \frac{\tanh\;\left(n\;\cdot \;a_{r}\right)}{\left(n\;\cdot \;a_{r}\right)}\right\}$
Cox	$E_{c}=\varphi_{m}E_{m}+\varphi_{f}E_{f}\cdot \left(1-\frac{\tanh\;\left(n\;\cdot \;a_{r}\right)}{\left(n\;\cdot \;a_{r}\right)}\right)$
Halpin-Tsai	$E_{c}=\frac{3}{8}E_{l}+\frac{5}{8}E_{t}$
